# Expression levels of chemokine (C-X-C motif) ligands CXCL1 and CXCL3 as prognostic biomarkers in rectal adenocarcinoma: evidence from Gene Expression Omnibus (GEO) analyses

**DOI:** 10.1080/21655979.2021.1952772

**Published:** 2021-07-16

**Authors:** Qi-yuan Lv, Hai-zhou Zou, Yu-yan Xu, Zhen-yong Shao, Ruo-qi Wu, Ke-jie Li, Xia Deng, Dian-na Gu, Hong-xiao Jiang, Meng Su, Chang-lin Zou

**Affiliations:** a Department of Radiotherapy, The First Affiliated Hospital of Wenzhou Medical University, Wenzhou, Zhejiang, China; b Department of Oncology, Wenzhou Hospital of Traditional Chinese Medicine, Wenzhou, Zhejiang, China; c Department of Medical Oncology, The First Affiliated Hospital of Wenzhou Medical University, Wenzhou, Zhejiang, China; d Omigen, Inc, Hangzhou, PR China

**Keywords:** Rectal adenocarcinoma, bioinformatics analysis, CXCL1, CXCL2, CXCL3

## Abstract

Rectal cancer is a life‑threatening disease worldwide. Chemotherapy resistance is common in rectal adenocarcinoma patients and has unfavorable survival outcomes; however, its related molecular mechanisms remain unknown. To identify genes related to the initiation and progression of rectal adenocarcinoma, three datasets were obtained from the Gene Expression Omnibus database. In total, differentially expressed genes were analyzed from 294 tumor and 277 para-carcinoma samples from patients with rectal cancer. Gene Ontology and Kyoto Encyclopedia of Genes and Genomes functions were investigated. Cytoscape software and MicroRNA Enrichment Turned Network were applied to construct a protein-protein interaction network of the dependent hub genes and related microRNAs. The Oncomine database was used to identify hub genes. Additionally, Gene Expression Profiling Interactive Analysis was applied to determine the RNA expression level. Tumor immune infiltration was assessed using the Tumor Immune Estimation Resource database. The expression profiles of hub genes between stages, and their prognostic value, were also evaluated. During this study, data from The Cancer Genome Atlas were utilized. In rectal adenocarcinoma, four hub genes including CXCL1, CXCL2, CXCL3, and GNG4 were highly expressed at the gene and RNA levels. The expression of CXCL1, CXCL2, and CXCL3 was regulated by has-miR-1-3p and had a strong positive correlation with macrophage and neutrophil. CXCL2 and CXCL3 were differentially expressed at different tumor stages. High expression levels of CXCL1 and CXCL3 predicted poor survival. In conclusion, the CXCL1 and CXCL3 genes may have potential for prognosis and molecular targeted therapy of rectal adenocarcinoma.

## Graphical abstract

**Figure:**
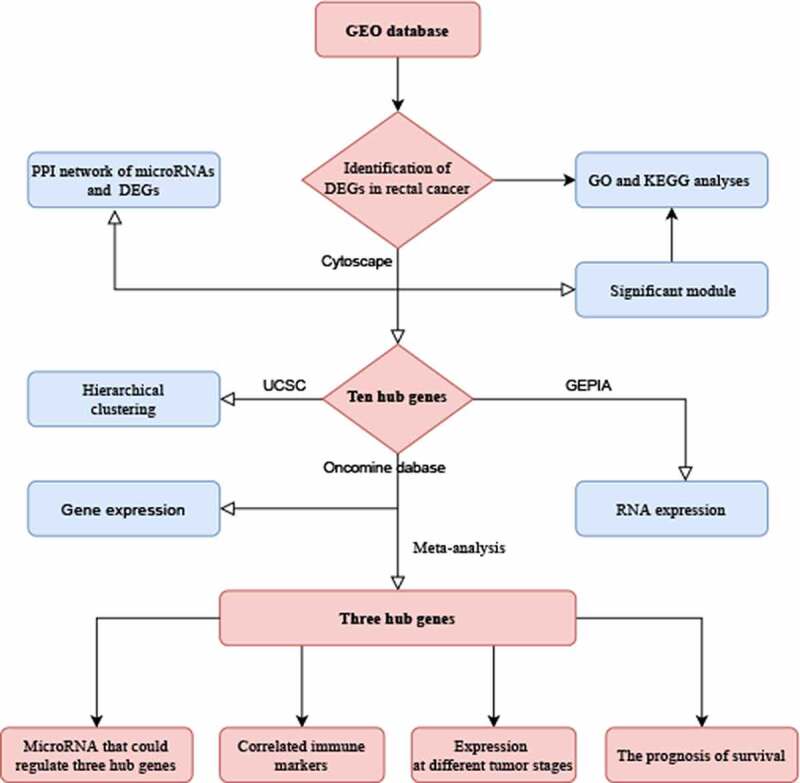

## 1. Introduction

Colorectal cancer (CRC) is the third most common cancer among males and females in America [1], and the fifth most common malignant tumor in Chinese males and females [2]. With extensive use of colonoscopy, the occurrence rate of rectal cancer has declined in the past few years. However, the early detection rate of rectal cancer did not improve significantly [1]. From the data provided by the National Center for Health Statistics, it can be predicted that, in 2020, around 147,950 people will be diagnosed with CRC and the number of deaths will reach 53,200, including 3,640 deaths in patients aged under 50 [3]. As rectal cancers exhibit an increasing and younger trend, early diagnosis is important for improving the outcome of the disease. Hence, additional efforts need to be taken to identify new biomarkers of rectal adenocarcinoma (READ), which could lead to the development of more effective treatment approaches.

Network medicine, a novel tool used to systematically explore a particular disease, has evolved rapidly in recent years [4,5]. Based on Gene Expression Omnibus (GEO) or The Cancer Genome Atlas (TCGA) database, the internal links between genes and diseases were better identified through network analysis [6,7]. For example, Grimaldi et al. used a network-based approach to explore the pathologic mechanisms of different clinicopathologic types of breast cancer, which could accurately discover the potential treatment targets [8]. Similarly, Panebianco et al. found that prostate cancer screening research could benefit from network medicine approach as it could facilitate the sharing of ideas among clinicians and data analysts [9]. These findings suggest that human diseases are rarely caused by a single molecular determinant, but more likely influenced by a network of interacting genes with the propensity to cluster together in the biological network. Hence, the combination of the network medicine and the GEO database to explore the role of genes in tumor diseases holds great promise.

Gene profiles using gene chips have been generally utilized to select differentially expressed genes (DEGs). Studies have indicated that certain genes in the colon and rectal cancer are associated with widespread changes, both at the mRNA and protein levels, which may offer a novel paradigm to better understand cancer biology [10]. READ is a disease with heterogeneous sensitivity to chemotherapy [11]. While biomarkers associated with treatment response and prognosis of READ patients have been explored, the findings are sometimes inconsistent [12–14]. Furthermore, chemotherapy resistance is common in READ patients and correlates with unfavorable progression free survival and overall survival (OS) [15], but the molecular mechanisms underlying treatment resistance are largely unknown.

The purpose of this study was to identify biomarkers that could predict the prognosis of READ to help patients obtain better survival. To identify potential biomarkers for READ, raw microarray data (GSE90627, GSE87211, and GSE68204) from the GEO database was analyzed using a variety of bioinformatic approaches. DEGs were determined by comparing the tumor to the para-cancerous tissue. Using several bioinformatics analysis methods to progressively screen for important genes, we focused on up- or down-regulated genes in rectal cancer samples and explored the biological significance of these genes. The biological functions of DEGs were determined by Gene Ontology (GO) annotation and Kyoto Encyclopedia of Genes and Genomes (KEGG) pathways analyses. A protein–protein interaction (PPI) network for the DEGs was utilized to select hub genes and those which correlated with pathogenesis and prognosis of READ patients were validated in different databases, including Oncomine and TCGA. We hypothesized that some microRNAs can target and link certain genes to participate in onset and progression of READ. Moreover, the association between immune cells and the expression of hub genes may further influence the occurrence and development of tumors. Ultimately, we hope to compare hub genes at different tumor stages and identify genes associated with OS of READ.

## 2. Materials and methods

### 2.1. Data source

The three microarray datasets were used in the present work. They were all downloaded from the GEO database (https://www.ncbi.nlm.nih.gov/geo/) [16]. Next, 2570 series about human rectal carcinoma were obtained. After the rational screening, three representative datasets (GSE90627, GSE87211, and GSE68204) were selected for analysis. The GSE90627 dataset contained 32 rectal cancer tissue samples and 96 normal samples, GSE87211 contained 203 rectal cancer samples and 160 noncancerous samples, and GSE68204 included 59 rectal cancer samples and 21 para-carcinoma samples. Due to the bioinformatics feature, ethical approval of this work was waived.

### 2.2. Data processing of differentially expressed genes (DEGs)

GEO2R, a freely available online tool (https://www.ncbi.nlm.nih.gov/geo/geo2r/) [16], was used to determine the DEGs from malignant rectal tumor and corresponding normal tissues. Then, the false discovery rate (FDR) and |logFC| were calculated. A |logFC| > 2 and FDR < 0.05 was considered statistically significant. The volcano map drawn by R language software was used to visualize the results. Each dataset was analyzed statistically, and the important overlap was presented via a Venn diagram networking tool (http://bioinformatics.psb.ugent.be/webtools/Venn/). The MIENTURNET platform (http://userver.bio.uniroma1.it/apps/mienturnet/) enables the extraction of the microRNAs that could target a list of genes provided as inputs [17]. Based on this platform, we identified the microRNAs which target DEGs with the default settings on the site, and the PPI network was performed by the Cytoscape software.

### 2.3. DEGs function annotation

The Database for Annotation, Visualization, and Integrated Discovery (DAVID; http://david.ncifcrf.gov) was utilized to describe the functions and signaling pathways of these DEGs [18]. GO function analysis (biologic processes [BPs], cellular components [CCs], and molecular functions [MFs]) was conducted to provide information about gene functions. The GO and KEGG enrichments were carried out. The analysis of biological functions and pathways related to DEGs was according to the DAVID. FDR < 0.05 was considered statistically significant.

### 2.4. Integration of protein–protein interaction (PPI) network and module analysis

The Search Tool for the Retrieval of Interacting Genes (STRING) database, (http://string-db.org/) containing 24,584,628 proteins from 5,090 organisms, is used to predict protein-protein interactions [19]. To assess the potential PPI relationship, we mapped DEGs to the STRING database. All parameters were set to default. The PPI pairs were collected with an interaction score > 0.4. Cytoscape software (https://cytoscape.org/) was applied to the PPI network’s visualization [20]. Highly connected nodes are often more important to maintain the entire network’s stability. Molecular Complex Detection (MCODE) is a plugin of Cytoscape. It is often applied to locate densely connected regions via a clustering algorithm. In this work, MCODE was utilized to find the most significant PPI networks’ model. Selection criteria were as follows: MCODE scores ≥ 5, cutoff of degree = 2, cutoff of node score = 0.2, Max depth = 100, and k-score = 2. Similarly, the GO and KEGG were used to analyze the genes in this module.

### 2.5. Hub genes selection and analysis

A highly connected protein node was screened via the Cytoscape plug-in cytoHubba [21]. According to the ranks of degree, the top ten genes were determined as the hub genes. The hub genes’ hierarchical clustering was constructed via UCSC Cancer Genomics Browser (http://genome-cancer.ucsc.edu) [22]. The Oncomine database’s (https://www.oncomine.org) Gaedcke Colorectal Statistics was utilized to analyze hub genes expression in READ [23]. Rectal tissue samples versus tumor samples were filtered according to analytic types, for example, Student’s *t* test for independent samples, fold change with genes ranking the top 20% as the threshold of significance. The minimum 10^th^, 25^th^, 90^th^, and maximum percentage data for each gene in normal and READ samples were drawn. The hub genes’ RNA expression level in samples was determined via Gene Expression Profiling Interactive Analysis (GEPIA) (http://gepia.cancer-pku.cn/index.html) [24]. The data are based on the TCGA database and Genotype-Tissue Expression (GTEx) projects [24]. Moreover, using the Gaedcke Colorectal Statistics and two additional datasets from Oncomine website, we further verified the discrepancy in hub genes’ expression between READ and para-carcinoma tissue. In addition, Tumor IMmune Estimation Resource (TIMER) database (https://cistrome.shinyapps.io/timer/) was utilized to assess the relationship between immune Cells and hub genes [25]. Similarly, the association of tumor staging and hub genes was further explored in GEPIA. Furthermore, TCGAportal (http://www.tcgaportal.org) was applied to analyze the hub genes’ OS. Finally, we used data from GSE87211 to verify the OS of the hub genes, and a figure was drawn by the Online consensus Survival for colorectal Cancer (http://bioinfo.henu.edu.cn/CRC/CRCList.jsp).

## 3. Results

CXCL1 and CXCL3, which were selected carefully from the GEO database, were the independent prognosis factors for rectal cancer. Results from a variety of bioinformatics analyses suggest that hub genes (CXCL1, CXCL2, and CXCL3) could be significant biomarkers for the prognosis of rectal cancer. It has been found that has-miR-1-3p could be involved in the progression of rectal cancer by regulating CXCL1, CXCL2, and CXCL3. In addition, these three hub genes had a strong positive correlation with macrophage and neutrophil and showed differential expression at different tumor stages. Data from TCGA and GEO verified that overexpression of gene CXCL1 and CXCL3 portends unfavorable survival outcomes in patients with rectal adenocarcinoma.

### 3.1 Identification of DEGs in rectal cancer

**Figure 1.**
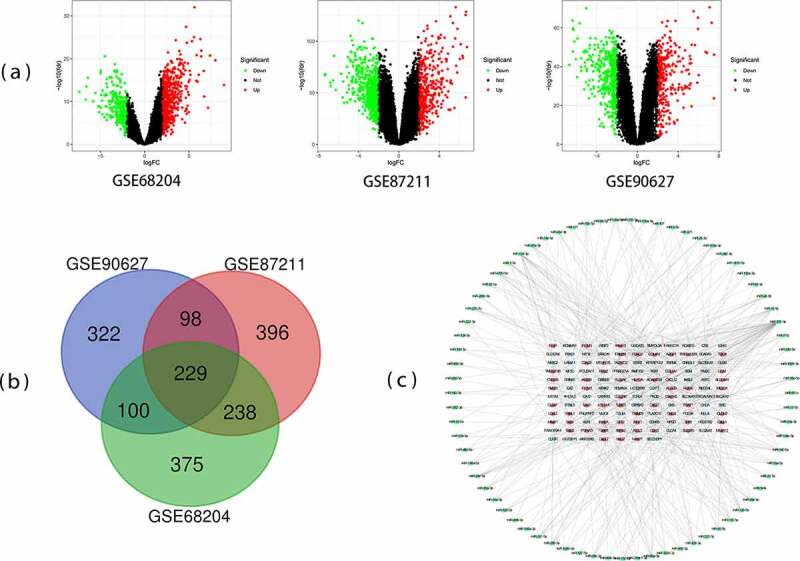
Differentially expressed genes (DEGs) from the three datasets. (a) The volcano plots of genes expression in GSE68204, GSE87211, and GSE90627. (b) Venn chart of DEGs shared between three GEO datasets (GSE68204, GSE87211, and GSE90627). (c) Protein-protein interaction (PPI) network of microRNA and target DEGs. Blue = downregulated DEGs; Red = upregulated DEGs; Green = microRNA

The volcano plots of three databases are shown in Figure 1(a). By comparing rectal cancer tissues with non-cancerous tissues samples, 942, 961, and 749 DEGs were identified in GSE68204, GSE87211, and GSE90627, respectively. As shown in Figure 1(b), the 229 DEGs overlapping among the three datasets comprised 144 downregulated genes and 85 upregulated genes. In the gene chip GSE68204, among 942 DEGs, 640 were upregulated and 302 were downregulated. From the GSE87211, 961 DEGs, including 563 downregulated genes and 398 upregulated, were identified. A total of 749 DEGs were found in GSE90627, of which 438 were down-regulated and 311 were up-regulated genes.

### 3.2 Functional enrichment analyses of DEGs

The functional categorization of DEGs was investigated via GO and KEGG analyses based on DAVID (Table 1). The GO categories consisted of three domains: BP, CC, and MF. The GO analysis revealed that the genes’ accumulation mostly occurred in BPs, such as bicarbonate transport, one-carbon metabolic process, and digestion. MF analysis suggested that the enrichment of genes principally occurred in carbonate dehydratase activity, CXCR chemokine receptor binding, metalloendopeptidase activity, hormone activity, chemokine activity, growth factor activity, and zinc ion binding. For the cell component, the enrichment of DEGs occurred in apical plasma membrane, proteinaceous extracellular matrix, extracellular space, extracellular exosome, extracellular region, extracellular matrix, microvillus membrane, basolateral plasma membrane, anchored component of membrane, and basement membrane. From the KEGG enrichment results, DEGs were basically enriched in pathways in pancreatic secretion, nitrogen metabolism, mineral absorption, steroid hormone biosynthesis, and cytokine-cytokine receptor interaction.

**Table 1.** Significantly enriched GO terms and KEGG pathways of DEGs

Category	Term	Description	Count	FDR
BP_DIRECT	GO:0015701	bicarbonate transport	10	2.96E-06
BP_DIRECT	GO:0006730	one-carbon metabolic process	8	3.47E-05
BP_DIRECT	GO:0007586	digestion	7	0.04596315
CC_DIRECT	GO:0005615	extracellular space	50	4.30E-10
CC_DIRECT	GO:0070062	extracellular exosome	61	4.53E-04
CC_DIRECT	GO:0005576	extracellular region	40	0.001135314
CC_DIRECT	GO:0005578	proteinaceous extracellular matrix	14	0.001135314
CC_DIRECT	GO:0016324	apical plasma membrane	14	0.002129044
CC_DIRECT	GO:0031012	extracellular matrix	13	0.008403893
CC_DIRECT	GO:0031528	microvillus membrane	4	0.03695261
CC_DIRECT	GO:0016323	basolateral plasma membrane	9	0.03695261
CC_DIRECT	GO:0031225	anchored component of membrane	7	0.049664957
CC_DIRECT	GO:0005604	basement membrane	6	0.049664957
MF_DIRECT	GO:0004089	carbonate dehydratase activity	6	1.53E-04
MF_DIRECT	GO:0045236	CXCR chemokine receptor binding	5	4.26E-04
MF_DIRECT	GO:0004222	metalloendopeptidase activity	9	0.007705725
MF_DIRECT	GO:0005179	hormone activity	8	0.012098268
MF_DIRECT	GO:0008009	chemokine activity	6	0.020729853
MF_DIRECT	GO:0008083	growth factor activity	9	0.040942654
MF_DIRECT	GO:0008270	zinc ion binding	28	0.040942654
KEGG_PATHWAY	hsa00910	Nitrogen metabolism	6	5.11E-04
KEGG_PATHWAY	hsa04972	Pancreatic secretion	8	0.021615885
KEGG_PATHWAY	hsa04978	Mineral absorption	6	0.021615885
KEGG_PATHWAY	hsa04060	Cytokine-cytokine receptor interaction	12	0.034663269
KEGG_PATHWAY	hsa00140	Steroid hormone biosynthesis	6	0.046299186

### 3.3 Integration of PPI network and module analysis

**Figure 2.**
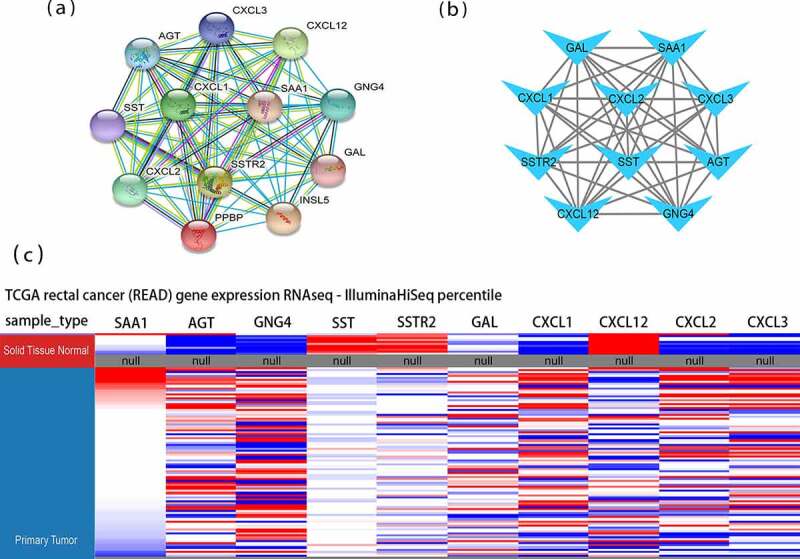
Protein-protein interaction (PPI) networks and hierarchical clustering analysis of hub genes. (a) Module 1 consisted of 12 nodes and 66 edges. (b) Hub genes’ PPI network was obtained using the MCC algorithm in the cytoHubba tool kits. (c) Utilizing UCSC construct hub genes’ hierarchical clustering. Blue: up-regulated; Red: down-regulated. MCC: Maximal Clique Centrality

To examine the interactions among the 229 DEGs, protein interactions were performed in STRING. We identified the potential microRNAs associated with DEGs and constructed a protein network of microRNAs and their target genes. The final PPI network contained 201 nodes and 386 edges (Figure 1(c)). According to MCODE, a plug-in from Cytoscape, the cluster having 12 nodes and 66 edges, including GNG4, CXCL12, AGT, CXCL3, PPBP, SSTR2, SST, CXCL2, SAA1, CXCL1, INSL5, and GAL, exhibited the highest score, indicating the highest degree of connectivity (Figure 2(a)). DAVID was used to functionally analyze the DEGs and the results suggested that genes from the present module were considerably enriched in immune response, extracellular region, CXCR chemokine receptor binding, and chemokine signaling pathway (Table 2).

**Table 2.** GO and KEGG pathway enrichment analyses of DEGs in the most significant module

Category	Term	Description	Count	FDR
BP_DIRECT	GO:0070098	chemokine-mediated signaling pathway	5	1.74E-05
BP_DIRECT	GO:0007186	G-protein coupled receptor signaling pathway	8	3.09E-05
BP_DIRECT	GO:0006954	inflammatory response	6	1.45E-04
BP_DIRECT	GO:0090023	positive regulation of neutrophil chemotaxis	3	0.004113001
BP_DIRECT	GO:0006955	immune response	5	0.004113001
BP_DIRECT	GO:0032496	response to lipopolysaccharide	4	0.004369455
BP_DIRECT	GO:0042127	regulation of cell proliferation	4	0.005346894
BP_DIRECT	GO:0060326	cell chemotaxis	3	0.016721759
BP_DIRECT	GO:0030593	neutrophil chemotaxis	3	0.016721759
CC_DIRECT	GO:0005576	extracellular region	10	2.70E-07
CC_DIRECT	GO:0005615	extracellular space	9	1.06E-06
MF_DIRECT	GO:0045236	CXCR chemokine receptor binding	5	3.31E-10
MF_DIRECT	GO:0008009	chemokine activity	5	2.75E-07
MF_DIRECT	GO:0008083	growth factor activity	4	0.001217509
MF_DIRECT	GO:0005179	hormone activity	3	0.011023041
KEGG_PATHWAY	hsa04062	Chemokine signaling pathway	6	1.80E-05
KEGG_PATHWAY	hsa04060	Cytokine-cytokine receptor interaction	5	0.001188554
KEGG_PATHWAY	hsa05134	Legionellosis	3	0.013693281
KEGG_PATHWAY	hsa05132	Salmonella infection	3	0.024012984
KEGG_PATHWAY	hsa04668	TNF signaling pathway	3	0.03156819

### 3.4 Identification and exploration of hub genes

According to the degree score evaluated by the Cytohubba plugin [21], the ten top-ranked genes, including SAA1, AGT, GNG4, SST, SSTR2, GAL, CXCL1, CXCL12, CXCL2, and CXCL3, were recognized as potential hub genes (Figure 2(b)). Hierarchical clustering revealed that these ten genes could generally distinguish the rectal tumor tissues from the noncancerous tissues (Figure 2(c)). Seven hub genes, including SAA1, AGT, GNG4, GAL, CXCL1, CXCL2, and CXCL3, were upregulated in rectal cancer tissues. In addition, three hub genes, including SST, SSTR2, and CXCL12, were significantly downregulated in the rectal cancer tissues. These hub genes had the potential to differentiate rectal cancer and normal tissues.

### 3.5 Expression of hub genes in READ

**Figure 3.**
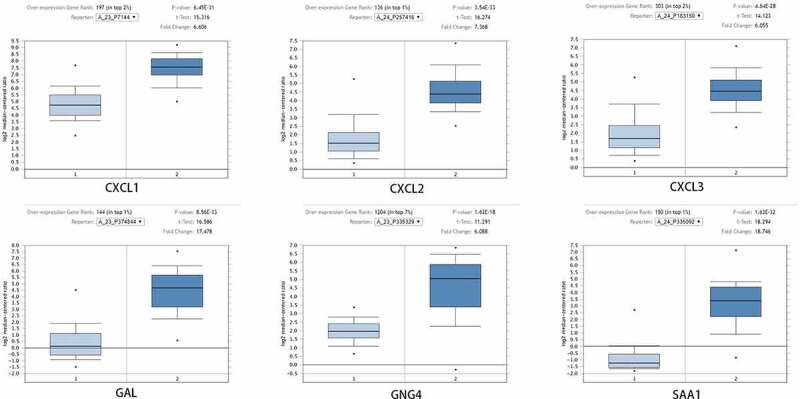
After Oncomine database analysis of the ten hub genes, six hub genes were differentially expressed in READ and para-carcinoma tissue. The expression of CXCL1, CXCL2, CXCL3, GAL, GNG4, and SAA1 were plotted to draw the boxplot; 1: rectum samples (n = 65); 2: READ samples (n = 65). READ: rectal adenocarcinoma

**Figure 4.**
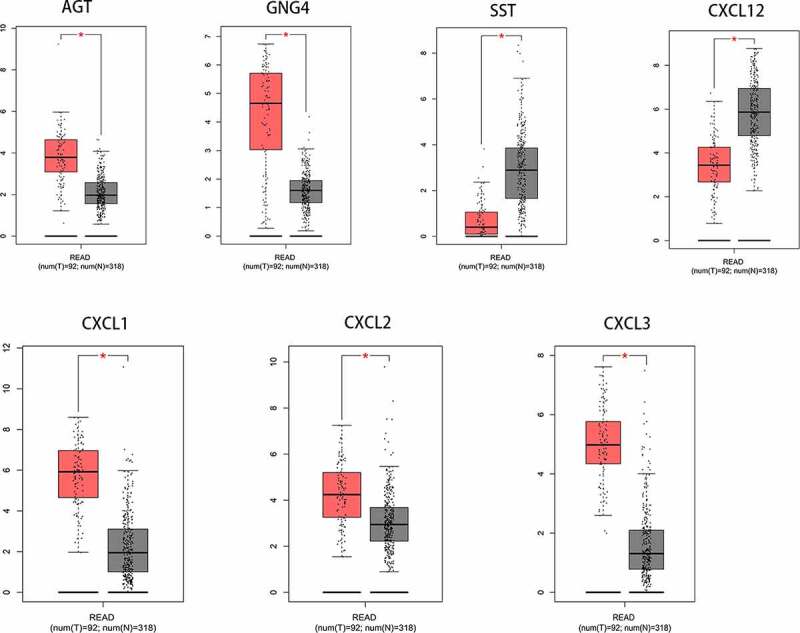
After analysis of the ten hub genes, the RNA expression levels of seven hub genes in tumor tissues and para-carcinoma tissues were significantly different. Data were obtained from TCGA and the GTEx projects (gray: normal; red: tumor). *p-value < 0.01. READ: rectal adenocarcinoma

To study the differential expression of hub genes in READ, we analyzed these ten genes in 65 READ samples and 65 noncancerous samples, which were available from the public Oncomine database. It was observed that the expression levels of GAL, GNG4, SAA1, CXCL1, CXCL2, and CXCL3 were notably different between READ samples and para-carcinomal rectum samples (P < 0.05) (Figure 3). According to the TCGA database and the GTEx projects, the RNA expression levels of AGT, GNG4, SST, CXCL12, CXCL1, CXCL2, and CXCL3 genes were statistically different between the READ and normal tissues (Figure 4). These results suggested that CXCL1, CXCL2, CXCL3, and GNG4 probably serve an indispensable role in the progression of READ. However, additional experimental validations are required to exploit the functions of these genes in READ.

### 3.6 Meta-analysis of three hub genes and their associations with the immune environment, tumor stages, and survival in READ patients

**Figure 5.**
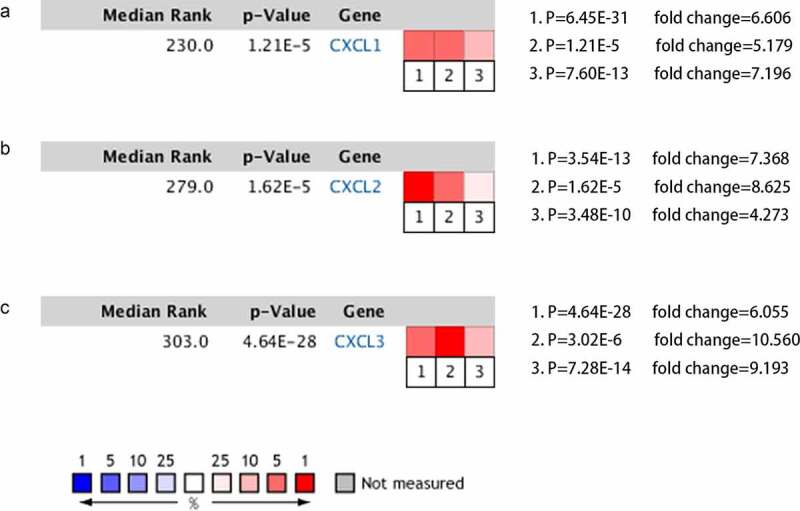
The gene expression levels of CXCL1, CXCL2, and CXCL3 were evaluated in cancer vs. normal tissue in three studies using Oncomine analysis. 1. Rectal Adenocarcinoma vs. Normal Gaedcke Colorectal, Genes Chromosomes Cancer,2010. 2. Rectal Adenocarcinoma vs. Normal Kaiser Colon, Genome Biol, 2007. 3. Rectal Adenocarcinoma vs. Normal TCGA Colorectal, No Associated Paper, 2011

**Figure 6.**
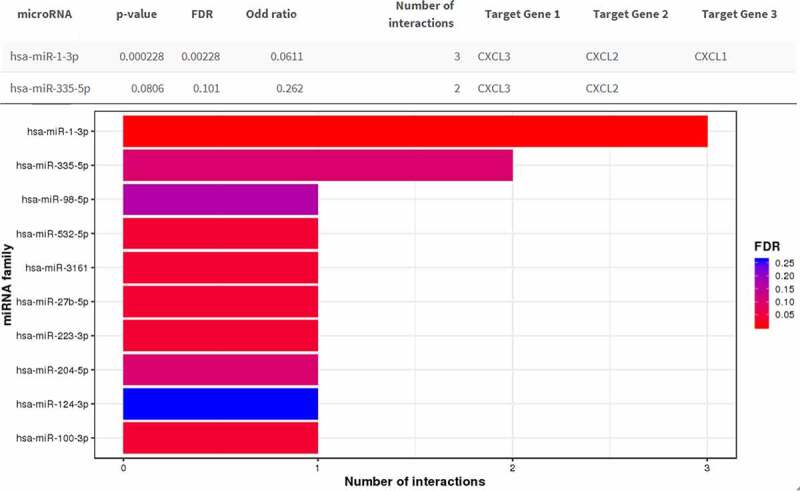
Result of the miRNA enrichment analysis from the MIENTURNE web tool. CXCL1, CXCL2, and CXCL3 were analyzed and hsa-miR-1-3p was significantly associated with these three genes (FDR < 0.01)

**Figure 7.**
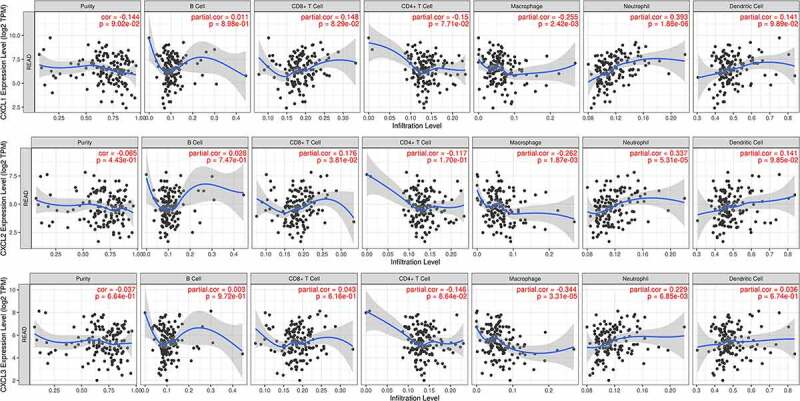
Correlation analyses between the expression of three hub genes and immune markers. The scatter plots of CXCL1, CXCL2, and CXCL3 with tumor purity, B cell, CD8 + T cell, CD4 + T cell, macrophage, neutrophil, and dendritic cell in READ were respectively drawn by the TIMER online database. There was no obvious association between the expression levels of CXCL1, CXCL2, and CXCL3 and infiltration of B cell, CD4 + T cell, CD8 + T cell, or dendritic cell. Likewise, no statistical correlation was seen between levels of CXCL1, CXCL2, and CXCL3 and tumor purity, whereas the expression of CXCL1, CXCL2, and CXCL3 was significantly positively correlated with macrophage and neutrophil. READ: rectal adenocarcinoma

**Figure 8.**
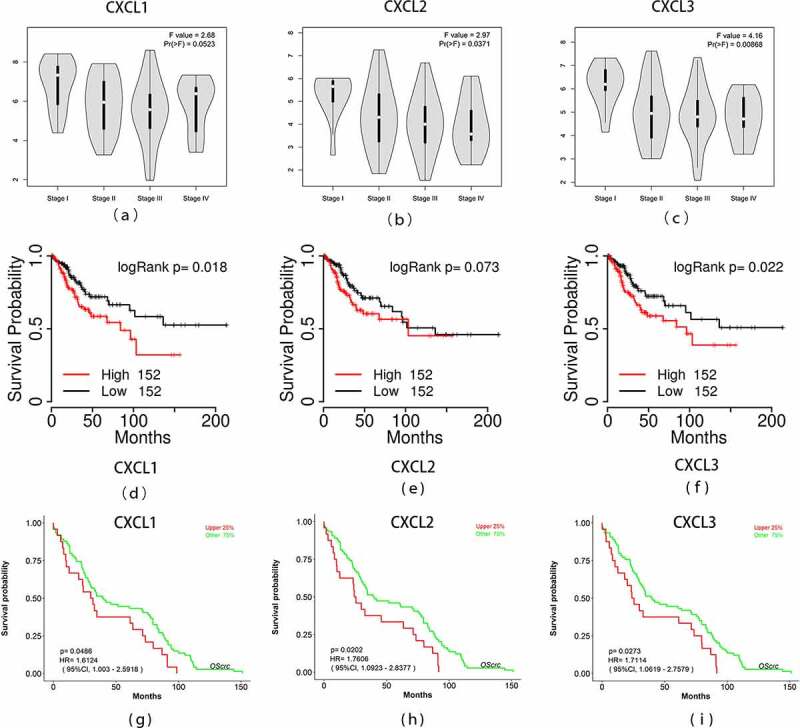
Overall survival analyses and the expression level of three hub genes at different stages. (a)-(c) The expression levels of the three hub genes in READ tissues at different stages was drawn by the GEPIA2 online database. Statistical significance was assessed using analysis of variance. Pr (>F) < 0.05 was accepted as statistically different. (d)-(f) Overall survival analyses of three hub genes were estimated by online tool TCGAPortal. (g)-(i) Overall survival analyses of three hub genes in GSE87211. Statistical significance: p-value < 0.05. READ: rectal adenocarcinoma

By analyzing the three datasets in the Oncomine database, the expression levels of CXCL1, CXCL2, and CXCL3 were higher in READ samples than in noncancerous tissues (Figure 5). Then, we identified the microRNAs that target these three hub genes (Figure 6). The fact that has-miR-1-3p targets three hub genes simultaneously (FDR <0.05) indicated that has-miR-1-3p could be involved in the progression of rectal cancer by regulating CXCL1, CXCL2, and CXCL3. Moreover, the expression of all three hub genes showed a significant positive correlation with macrophages and neutrophils (Figure 7), which suggested that hub genes may promote the development and occurrence of READ by mobilizing and regulating the immune cells. The results of the analysis of the three genes across tumor stages are presented in Figure 8(a-c)). It was observed that the gene expression levels of CXCL2 and CXCL3 were significantly different in READ patients at different stages. According to the TCGAportal, READ patients with up-regulated CXCL1, CXCL2, and CXCL3 showed poor OS (log-rank p = 0.018, 0.073, and 0.022 for CXCL1, CXCL2, and CXCL3, respectively (Figure 8(d-f)). As shown in Figure 8(g-i), the OS of READ patients with up-regulated CXCL1, CXCL2, and CXCL3 was worse (p-value = 0.0486, 0.0202, and 0.0273 for CXCL1, CXCL2, and CXCL3, respectively) based on the data from GSE87211.

## 4. Discussion

In this study, based on the three datasets (GSE90627, GSE87211, and GSE68204), 229 DEGs were selected in rectal cancer samples, including 85 up-regulated genes and 114 down-regulated genes. They had a connection with biological processes such as bicarbonate transport, one-carbon metabolic process, and digestion, and significantly enriched in pathways involved in nitrogen metabolism, pancreatic secretion, mineral absorption, steroid hormone biosynthesis, and cytokine-cytokine receptor interaction. According to the PPI network, we subsequently selected the top ten DEGs as hub genes. Furthermore, the Oncomine microarray database and TCGA RNA sequencing database were used for further study of these genes. The differential expression of six genes (CXCL1, CXCL2, CXCL3, GAL, GNG4, and SAA1) in READ was validated in an Oncomine dataset. Furthermore, the RNA expression levels of AGT, GNG4, SST, CXCL12, CXCL1, CXCL2, and CXCL3 genes were significantly different of READ in the TCGA dataset. Thus, CXCL1, CXCL2, CXCL3, and GNG4 genes were consistently and significantly differentiated expressed at both the gene and RNA expression levels. By comparing data from the Oncomine database for two additional studies on READ, we observed the differential expression levels of CXCL1, CXCL2, and CXCL3 genes in all three datasets. Subsequently, we subjected these three genes to KEGG analyses and obtained similar results as those obtained in the significant module selected by Cytoscape. From the KEGG enrichment results, three hub genes were significantly enriched in the following pathways: Chemokine signaling pathway, Cytokine-cytokine receptor interaction, Legionellosis, Salmonella infection, and TNF signaling pathway.

Upon further exploration, we found that all three hub genes were involved in has-miR-1-3p. Recent studies have shown that has-miR-1-3p is involved in the regulation of a variety of cancers. For example, Jiao et al. found that has-miR-1-3p blocks epithelial-mesenchymal transformation in lung cancer by regulating c-Met [26]. In gastric cancer and bladder cancer, the expression of has-miR-1-3p has been reported to inhibit the proliferation and invasion of cancer cells by targeting a certain gene or protein [27,28]. All these evidence suggest that has-miR-1-3p could inhibit tumor growth by targeting a gene. In our study, we contend that has-miR-1-3p may affect the prognosis of rectal cancer by targeting CXCL1, CXCL2, and CXCL3. However, further studies are needed to validate this hypothesis.

Next, we pursued the relationship between these hub genes expression and immunocyte infiltration. Intriguingly, the expression of all three genes had a positive correlation with macrophages and neutrophils. Indeed, CXCL1 derived from tumor-associated macrophages proved to be an important factor in the promotion of breast cancer [29]. In bladder cancer, the production of CXCL1 in tumor-associated macrophages supported tumor implantation in the wall of the murine bladder [30]. A recent study showed that CXCL3 was highly upregulated in a certain macrophage and portended poor survival in pancreatic ductal adenocarcinoma [31]. Therefore, CXCL1 and CXCL3 may be involved in the progression of rectal cancer under the regulation of related macrophages.

Through comprehensive meta-analysis, Zhang et al. found that CXCL1 was often highly expressed in advanced tumors [32]. However, in this study, no significant differences were found in the CXCL1 gene between the different stages of rectal cancer and the expression levels of only CXCL2 and CXCL3 genes varied with the stages of READ. In a study by Chen et al., ZC3H12A gene expression significantly correlated with the expression of CXCL1, CXCL2, and CXCL3 had higher expression at the mRNA levels in the early stage of colorectal cancer [33].

In addition, the three hub genes’ survival analysis manifested that the two up-regulated genes, CXCL1 and CXCL3, were remarkably associated with the OS of READ patients according to the TCGA and GEO database. The expression levels of these two genes are promising biomarkers that have the potential to be used in predicting the prognosis of READ. First, these two genes were identified using three gene expression profiling datasets. Second, these genes were simultaneously validated in multiple authoritative databases. Third, these genes have been previously proved to be profoundly involved in different carcinomas.

There has been increasing evidence that chemokines have a critical role in carcinogenesis and disease progression. CXCL1 (C-X-C motif chemokine ligand 1) is a functional gene. In addition, it is a member of the chemokines’ CXC subfamily. Previous studies have revealed that CXCL1 is involved in the signaling axis [34], immune suppression [35], and chemoresistance of cancer cells [36]. Overexpression of CXCL1 has been found in several malignant tumors, such as pancreatic cancer [37], colorectal carcinoma [38], bladder cancer [30], liver cancer [39], and oral squamous carcinoma [40]. Upregulation of CXCL1 may be related to unfavorable prognosis in malignant diseases. It has been reported that chemokines from the CXC subfamily exert both pro-tumor or anti-tumor activity effects [41] and may be used as a biomarker of treatment responses and serve as drug targets in colorectal cancer [42]. By analyzing 99 clinical samples and human CRC cell lines, Ogawa et al. found that up-regulated expression of CXCL1 hindering the CXCL1/8-CXCR2 axis may offer a new method for the treatment of SMAD4-negative CRC [43]. CXCL2, an important paralog of gene CXCL1, is a protein coding gene. Previous studies have revealed that elevated expression of CXCL2 in tumor tissues is related to advanced stages and worse prognosis of bladder cancer [44]. Chen and colleagues found that CXCL1, CXCL2, and CXCL3 were all highly expressed in colon cancer tissues [45]. However, several other studies reported down-regulated CXCL2 expression in hepatocellular carcinoma, possibly due to methylation-related mechanism [46,47]. Similar to CXCL1 and CXCL2, CXCL3 also belongs to the CXC chemokine family. According to Liao et al., high expression of CXCL3, which binds to CXCR2 on myeloid-derived suppressor cells, accelerates their migration toward the tumor microenvironment [48]. Similarly, in a study by Ruan et al., high CXCL3 expression was related to increase in mortality in the subgroup of patients with colon cancer whose tumors were smaller than 5 cm [49]. These evidences suggest that CXCL1, CXCL2, and CXCL3 may be indirectly involved in the genesis and progression of carcinoma through the mobilization of human immune cells. In addition, their mechanisms may vary in different tumor stages.

Besides CXCL1, CXCL2, and CXCL3, we mined seven other hub genes related to rectal cancer, including SAA1, AGT, GNG4, SST, SSTR2, GAL, and CXCL12. Most of them have been reported to participate in the development of malignant tumors [50–54]. However, because of serious differences in the strictness of detection standards between different platforms, mRNA microarray systems available in the market cannot show ideal inter-platform concordance. The main limitation of this study is the absence of experimental work to validate the results obtained. Hence, additional studies are required to confirm the findings of our study.

## 5. Conclusions

In the present study, a total of 229 DEGs were identified via comprehensive bioinformatics analysis, which included 85 upregulated and 144 downregulated genes in READ. The results indicated that ten hub genes, named SAA1, AGT, GNG4, SST, SSTR2, GAL, CXCL1, CXCL12, CXCL2, and CXCL3, may play an indispensable role in the initiation and prognosis of READ. Among these hub genes, differential expression of CXCL1, CXCL2, and CXCL3 was confirmed through several databases. In addition, the association between these genes, microRNA, tumor-infiltrating immune cells and clinical stages were investigated. Furthermore, the expression levels of CXCL1 and CXCL3 were associated with READ prognosis. Our study identified potential biomarker genes for READ, providing clues for future research of targeted drug therapy.

## Data Availability

The datasets generated during and/or analyzed during the current study are available from the corresponding author on reasonable request.

## Research highlights

- In READ, the expression of CXCL1, CXCL2, and CXCL3 had a strong positive correlation with macrophage and neutrophil.
- Gene CXCL2 and CXCL3 had various expression levels in READ patients at different stages.
- Overexpression of CXCL1 and CXCL3 were found to have prognostic values with READ.
